# Increasing *p*CO_2_ correlates with low concentrations of intracellular dimethylsulfoniopropionate in the sea anemone *Anemonia viridis*

**DOI:** 10.1002/ece3.946

**Published:** 2014-01-21

**Authors:** Esther M Borell, Michael Steinke, Rael Horwitz, Maoz Fine

**Affiliations:** 1 The Interuniversity Institute for Marine SciencesEilat, 88000, Israel; 2 Coral Reef Research Unit, School of Biological Sciences, University of EssexColchester, CO4 3SQ, U.K; 3 The Mina and Everard Goodman Faculty of Life Sciences, Bar-Ilan UniversityRamat-Gan, 52900, Israel

**Keywords:** Chlorophyll, CO_2_ vent, DMSP, primary research article, protein, superoxide dismutase, zooxanthellae

## Abstract

Marine anthozoans maintain a mutualistic symbiosis with dinoflagellates that are prolific producers of the algal secondary metabolite dimethylsulfoniopropionate (DMSP), the precursor of the climate-cooling trace gas dimethyl sulfide (DMS). Surprisingly, little is known about the physiological role of DMSP in anthozoans and the environmental factors that regulate its production. Here, we assessed the potential functional role of DMSP as an antioxidant and determined how future increases in seawater *p*CO_2_ may affect DMSP concentrations in the anemone *Anemonia viridis* along a natural *p*CO_2_ gradient at the island of Vulcano, Italy. There was no significant difference in zooxanthellae genotype and characteristics (density of zooxanthellae, and chlorophyll a) as well as protein concentrations between anemones from three stations along the gradient, V1 (3232 *μ*atm CO_2_), V2 (682 *μ*atm) and control (463 *μ*atm), which indicated that *A. viridis* can acclimate to various seawater *p*CO_2_. In contrast, DMSP concentrations in anemones from stations V1 (33.23 ± 8.30 fmol cell^−1^) and V2 (34.78 ± 8.69 fmol cell^−1^) were about 35% lower than concentrations in tentacles from the control station (51.85 ± 12.96 fmol cell^−1^). Furthermore, low tissue concentrations of DMSP coincided with low activities of the antioxidant enzyme superoxide dismutase (SOD). Superoxide dismutase activity for both host (7.84 ± 1.37 U·mg^−1^ protein) and zooxanthellae (2.84 ± 0.41 U·mg^−1^ protein) at V1 was 40% lower than at the control station (host: 13.19 ± 1.42; zooxanthellae: 4.72 ± 0.57 U·mg^−1^ protein). Our results provide insight into coastal DMSP production under predicted environmental change and support the function of DMSP as an antioxidant in symbiotic anthozoans.

## Introduction

Dimethylsulfoniopropionate (DMSP) is a secondary metabolite that is produced and accumulated at high intracellular concentrations (typically hundreds of mmol L^−1^) by many microalgae (Keller et al. 1989) including members of the dinoflagellates (Caruana et al. 2012). It is the precursor of dimethylsulfide (DMS), the main natural source of reduced sulfur released to the atmosphere (Bates et al. 1987; Kettle and Andreae 2000). Despite controversy about its function in a negative feedback mechanism to global warming (Quinn and Bates 2011), the atmospheric oxidation products of DMS play a major role in the formation of clouds, cloud albedo, and thus in the regulation of global climate (Charlson et al. 1987; Simó 2001).

Cellular production of DMSP is complex, and concentrations are affected by a multitude of abiotic variables including salinity, nutrients, light, and temperature (Stefels 2000; Stefels et al. 2007). This is further complicated by a suite of biological processes governing the direct enzymatic cleavage of DMSP (Yost and Mitchelmore 2009), and bacterial (Johnston et al. 2008) and fungal (Kirkwood et al. 2009) degradation. High levels of atmospheric CO_2_ continue to increase aqueous *p*CO_2_ and result in the concomitant decrease in seawater pH (Caldeira and Wickett 2005), a phenomenon referred to as “ocean acidification” (OA). However, the effect of increasing *p*CO_2_ on intracellular DMSP concentration is poorly understood. Data from laboratory incubations with microalgae (760 *μ*atm; Avgoustidi et al. 2012) and field mesocosm experiments with a mixed natural phytoplankton population (750 *μ*atm; Hopkins et al. 2010) suggest a decrease in DMSP while several strains of the coccolithophore *Emiliania huxleyi* respond with an increase in intracellular DMSP under elevated *p*CO_2_ (790 and 1000 *μ*atm) and a 4 to 6°C increase in temperature (Spielmeyer and Pohnert 2012; Arnold et al. 2013). Similarly, varied is the response of DMSP to *p*CO_2_ in seaweeds; Kerrison et al. (2012) found no changes in DMSP in *Ulva* spp at 550–1250 *μ*atm, while Borell et al. (2013) reported an increase in DMSP in *Ulva lactuca* following exposure to 4000 *μ*atm.

Many marine organisms, especially those belonging to the anthozoans such as corals and sea anemones, maintain a mutualistic symbiosis with DMSP-producing dinoflagellates in the genus *Symbiodinium*, commonly known as “zooxanthellae” (Hill et al. 1995; Van Alstyne et al. 2009). These are thought to make a substantial contribution to the amount of DMS entering the atmosphere (Jones et al. 1994; Broadbent and Jones 2004; Jones and Trevena 2005). While the functional role of DMSP in macroalgae and free living phytoplankton is fairly well understood (e.g., Stefels 2000), significant questions remain regarding the physiological roles of DMSP in symbiotic anthozoans and the environmental factors that regulate its production. While current evidence suggests that DMSP in anthozoans is produced solely by the algal symbionts (Van Alstyne et al. 2009), high concentrations of DMSP are also found in the tissue of the coral host implying translocation of DMSP from symbiont to host, which indicates that DMSP furnishes an important role as secondary metabolite in both symbiotic partners (Broadbent et al. 2002; Yost et al. 2010). However, information on how either of the partners (i.e., animal vs. alga) affect specific DMSP concentrations is not available.

Symbiotic corals and anemones are frequently exposed to hyperoxic conditions within their tissues that lead to the production of harmful reactive oxygen species (ROS) (Dykens and Shick 1984; Lesser 2006) and DMSP, and its enzymatic breakdown products, DMS, acrylate, dimethyl sulfoxide, and methane sulfinic acid are potent scavengers of ROS (Sunda et al. 2002). Because high concentrations of DMSP were observed in stressed corals following thermal bleaching and exposure to ROS-inducing copper (Broadbent et al. 2002; Yost et al. 2010), DMSP has been proposed to function as an antioxidant in these organisms. While OA is expected to have adverse consequences for various biological processes (Kroeker et al. 2010), in anemones it may improve photosynthetic performance (Suggett et al. 2012; Towanda and Thuesen 2012) and result in a lessened production of ROS. In higher plants, for example, elevated concentrations of CO_2_ have been shown to decrease concentrations of antioxidant enzymes such as superoxide dismutase (SOD) (Schwanz et al. 1996), an important antioxidant enzyme in both anthozoan hosts and their symbiotic zooxanthellae (Lesser 2006).

Here, we investigated the total tissue concentrations of DMSP (i.e., DMSP in the tissue of both animal and algae) in the Mediterranean sea anemone *Anemonia viridis* along a natural CO_2_ seawater gradient that arises from a cold vent system along the shore of the island Vulcano, Italy. Our aims were to test (1) whether DMSP in *A. viridis* is sensitive to different CO_2_ concentrations along the gradient and (2) whether cellular DMSP concentrations are related to activity of SOD.

## Materials and Methods

**Figure 1:**
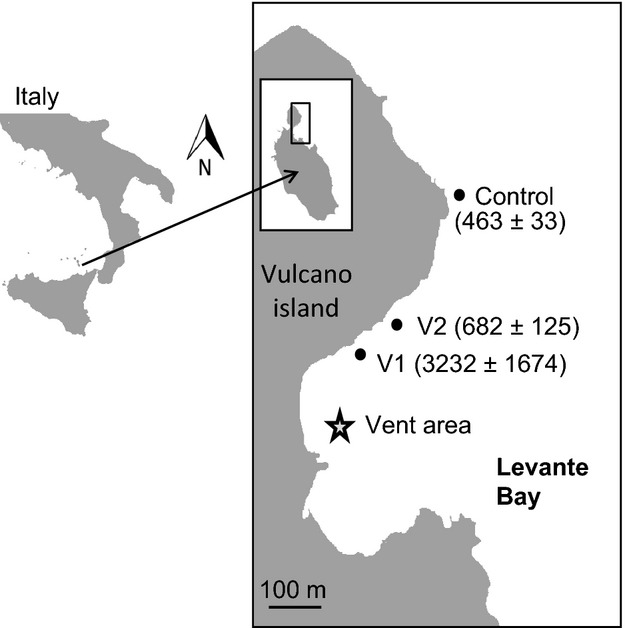
Map of the study area, Levante Bay (Vulcano island), Italy, showing the vent area and sampling stations V1, V2, and control. Data represent the calculated *p*CO_2_ (mean ± SD) in *μ*atm at each station (*n* = 4).

Samples were collected along the sublittoral in Levante Bay, North Vulcano Island (38°25′N, 14°57′E), NE of Sicily, Italy (Fig. 1) in May 2012. The Bay is located on the eastern side of the isthmus of Vulcano island and is characterized by the presence of submarine gas vents that release CO_2_ creating an extensive CO_2_/pH gradient that runs parallel to the northeastern coast of the island. This site has been used extensively as a natural laboratory for OA studies (Johnson et al. 2011; Arnold et al. 2012; Suggett et al. 2012; Boatta et al. 2013) as the average pH ranges from 6.05 to 8.29 at >350 m from the vent site (Johnson et al. 2011; Boatta et al. 2013) providing an environment representing possible future CO_2_ scenarios.

The sea anemone *A. viridis* is a dominant benthic organism in Levante Bay and becomes increasingly abundant with increasing *p*CO_2_ (Suggett et al. 2012). We selected three sampling stations compatible with previous studies (Johnson et al. 2011; Arnold et al. 2012; Suggett et al. 2012; Boatta et al. 2013) at ˜260 m (V1, high *p*CO_2_), ˜300 m (V2, intermediate *p*CO_2_), and ˜400 m (control) distance to the vents (Fig. 1). The stations were shallow (1–2 m), and sampling was carried out by snorkeling. Between 5 and 10, tentacles were clipped from each of 16 haphazardly selected anemones (oral disk size 2.5 to 3.5 cm) at every station using a pair of scissors. The samples were stored on ice, transported to the laboratory immediately, and frozen at −20°C in order to minimize the degradation of DMSP and antioxidant enzymes.

Hourly light recordings were carried out for three consecutive days at each station close to the seabed at a depth of 1–2 m with HOBO Pendant® Temperature/Light data loggers (Onset, Pocasset, MA). The logged light data (Table 1) were converted from lux to *μ*mol·m^2^·s^−1^ PAR (Table 1) (Thimijan and Heins 1983). The pH (NBS scale) together with temperature at each station was measured haphazardly once every day for 4 days (YSI Professional Plus, Handheld Multiparameter Instrument, Yellow Springs, OH), and water samples were taken and frozen at −20°C for measurements of total alkalinity (TA). TA was quantified with a Metrohm 862 compact titrosampler (Cohen 2011). The *p*CO_2_ levels were calculated from salinity (Johnson 2012), TA, and pH measurements using the program CO_2_SYS (Pierrot et al. 2006) and the constants of Mehrbach et al. (1973). Seawater parameters are shown in Table 1.

**Table 1:** Measurements of pH and TA taken at four consecutive days in May 2013, calculated *p*CO_2_ and mean midday (12:00–13:00 h) light intensities over three consecutive days along a *p*CO_2_ gradient at the island of Vulcano, Italy.

Station	pH (NBS scale)	TA (mmol·kg^−1^)	*p*CO_2_ (*μ*atm)	Light (*μ*mol·m^−2^·s^−1^)
V1	7.22	2.598	4808	588 ± 85
	7.24	2.573	4506	
	7.70	2.561	1468	
	7.54	2.487	2144	
V2	8.04	2.488	600	650 ± 71
	7.94	2.493	778	
	7.93	2.491	800	
	8.07	2.472	552	
Control	8.16	2.464	427	643 ± 24
	8.10	2.480	506	
	8.14	2.468	453	
	8.13	2.467	466	

There were no significant differences in light intensity (*n* = 21, ±SD) between the stations (*F*_2, 60_ = 1.62, *P* = 0.207). Seawater temperatures ranged from 18.5 to 19.5°C.

### Sample processing

Half of the tentacles were processed for DMSP, total protein, and zooxanthellae characteristics (i.e., chlorophyll a (chl a) and zooxanthellae densities) (*n* = 16) at the sampling site. Samples were weighed (CT 1202, Citizen, accuracy 0.01 g) and homogenized in sterile filtered seawater (FSW, 0.2 *μ*m) with an electric homogenizer (DIAX 100 homogenizer Heidolph Instruments GmbH & Co. KG, Schwabach, Germany). One milliliter of the homogenate was used directly for quantification of total DMSP (see below). The remaining homogenate was divided into subsamples before freezing these, and the remaining tentacles for SOD analyses and the determination of the zooxanthellae genotype at −20°C. The samples were then transported on dry ice to the Interuniversity Institute for Marine Sciences (IUI), Israel, where they were stored at −80°C pending analyses.

### Total protein

Total protein content was determined after Bradford (1976) using a commercial kit (Bio-Rad, Laboratories, Hercules, CA). In brief, for the protein extraction, 100 *μ*L of the tissue homogenate of each sample was sonicated on ice with a Branson Sonifier B12 (Branson Sonic Power Co., Danbury, Connecticut) for 20 sec and centrifuged at 2900 g for 20 min. Protein concentrations were assessed spectrophotometrically (Multiskan spectrum, Thermo Fisher Scientific Inc., Waltham, MA) at 595 nm using bovine albumin as the standard.

Zooxanthellae characteristics and genotype for measurements of chl a, zooxanthellae densities, and protein, 2 mL of homogenate was centrifuged (1900 g at 4°C) and resuspended four times in FSW. Resuspended zooxanthellae were used for chl *a* extraction in acetone (100%) at 4°C in the dark for 24 h. Concentrations were determined spectrophotometrically (Jeffrey and Humphrey 1975). Zooxanthellae densities were quantified from 4 replicate counts using a Neubauer haemocytometer. Zooxanthellae densities and chl *a* were normalized to protein concentration.

To determine the zooxanthellae genotypes, nucleic acid extractions (*n* = 5) were conducted using a modified Promega Wizard genomic DNA extraction protocol (LaJeunesse et al. 2003). Symbiont identity was characterized by denaturing gradient gel electrophoresis (DGGE) fingerprinting of the partial 5.8S and internal transcribed spacer (ITS) region 2 (LaJeunesse 2002). The region was amplified using a touch-down thermal cycle profile with the primers “ITS2clamp” and “ITSintfor2” (LaJeunesse and Trench 2000), and the PCR products resolved on denaturing gels (45–80% of 7 mol·L^−1^ urea & 40% formamide) using a CBS Scientific system (Del Mar, CA) for 16 h at 115 volts. The dominant band of the symbiont's DGGE profile was excised, reamplified, and cycle-sequenced to provide the ITS2 sequence that dominates the symbiont's genome.

### Quantification of DMSP_t_ and SOD

For the quantification of total DMSP (DMSP_t_ = sum of particulate and dissolved DMSP and DMS), 1 mL of homogenate was added to 2 mL 0.5 mol·L^−1^ NaOH in a gas-tight, screw cap headspace vial. This alkaline hydrolysis rapidly converts DMSP to equimolar concentrations of DMS, which can be quantified using gas chromatographic methods with direct injection of headspace (Steinke et al. 2011). Results were expressed as femtomole DMSP_t_ per zooxanthella cell and nmol DMSP_t_ per mg protein to allow a comparison with SOD activity (see below).

Samples for SOD measurements (*n* = 10) were homogenized in cold HEPES buffer pH 7.3 (4-(2-hydroxyethyl)-1-piperazineethanesulfonic acid; Biological Industries, 03-025-1B). A 2-mL subsample was taken for the analyses of protein concentrations and zooxanthellae densities. To separate the host from the zooxanthellae fraction, a 2-mL aliquot was centrifuged (2900 g for 20 min) and resuspended twice in HEPES buffer. The supernatant was used for analysis of animal SOD. Zooxanthellae contained in the pellet were resuspended in 0.5 mL of buffer and sonicated on ice for 20 sec. The zooxanthellae were centrifuged (2900 g for 20 min), and the supernatant was analyzed for algal SOD. The combined activities of three types of SOD (Cu/Zn-, Mn-, and Fe-SOD) in both animal host and zooxanthellae were determined spectrophotometrically at 450 nm using the Superoxide Dismutase Assay Kit (Cayman Chemical Company, Ann Arbor, MI) following manufacturer's instructions. In brief, SOD activity was assessed by measuring the dismutation of superoxide radicals generated by xanthine oxidase and hypoxanthine. One unit of SOD was defined as the amount of enzyme required for 50% inhibition of cytochrome *c* reduction. SOD activity for each sample was expressed as units (U) of enzyme activity per milligram protein.

### Data analyses

Data were checked for homogeneity of variances using the Cochran's *C*-test and analyzed using one-way ANOVA. The data for zooxanthellae densities were ln(x)-transformed prior to the analysis. Repeated measurements one-way ANOVA was used to determine whether there were significant differences in midday (12:00–13:00 h) light intensities between stations. Student–Newman–Keuls (SNK) tests were used for *post hoc* multiple comparisons. All data were analyzed using WinGMAV (EICC, University of Sydney, Australia).

## Results

There were no significant differences in chl *a* content (*F*_2, 45_ = 1.12, *P *=* *0.334), zooxanthellae densities (*F*_2, 45_ = 0.32, *P *=* *0.724), and protein concentrations (*F*_2, 45_ = 1.00, *P *=* *0.376) between anemones at the different stations. Chl a content (mg chl a mg^−1^ protein ± SE) varied between 3.8 ± 1.23 at the control station, 3.43 ± 1.5 and V2, and 4.41 ± 1.66 at V1. Zooxanthellae densities (cells mg^−1^ protein ± SE) averaged to 1.14 ± 0.18 × 10^6^ at the control site and decreased with increasing *p*CO_2_ to 1.1 ± 0.13 × 10^6^ at V2 and 0.94 ± 0.07 × 10^6^ at V1. Similarly, protein concentration (mg protein g^−1^ FW) was highest at the control site (39.87 ± 2.82) and decreased with increasing *p*CO_2_ to 38.09 ± 5.07 at V2 and 35.24 ± 5.37 at V1.

**Figure 2:**
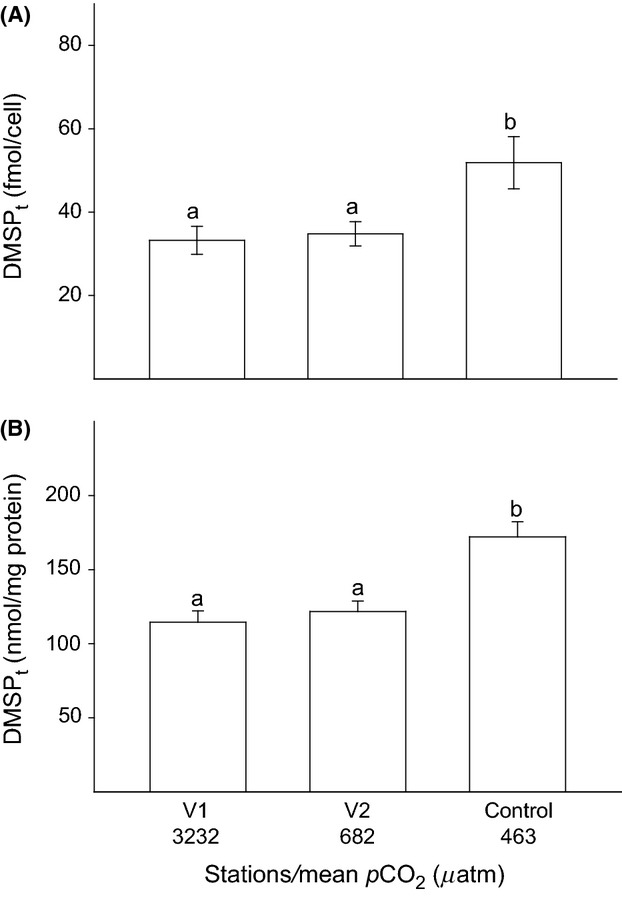
Mean DMSP_t_ concentration expressed as (A) fmol per cell and (B) nmol per mg of protein (*n* = 16, ±SE) in the tentacles of *Anemonia viridis* from three stations along the natural *p*CO_2_ gradient at Vulcano island. Letters above error bars indicate significant differences between groups (SNK).

**Figure 3:**
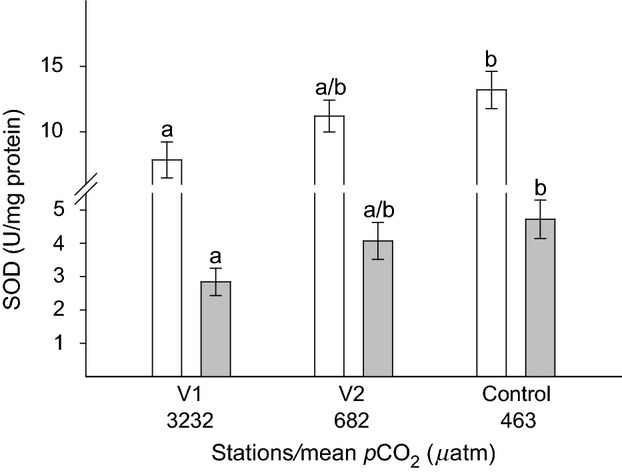
Mean SOD activity (*n* = 10, ±SE) in the tentacles of *Anemonia viridis* from three stations along the natural *p*CO_2_ gradient at Vulcano island. White bars show data for SOD activity in the anemone host and gray bars in the zooxanthellae. Letters above error bars indicate significant differences between groups (SNK).

There was no variation in zooxanthellae genotype. Anemones of all three stations were associated with symbionts of *Symbiodinum* type A19. *p*CO_2_ had a significant effect on DMSP_t_ concentrations in the tentacles of *A*. *viridis* both when normalized to cell (*F*_2, 45_ = 6.47, *P *=* *0.028; Fig. 2A) and to protein (*F*_2, 45_ = 13.96, *P *=* *0.000; Fig. 2B). Irrespective of normalization indices, DMSP_t_ concentrations from the control station were about 35% higher (51.85 ± 6.26 fmol cell^−1^ or 172.12 ± 10.13 nmol·mg^−1^ protein; equivalent to 6.49 ± 0.19 *μ*mol·g^−1^ fresh weight (FW)) than concentrations in tentacles from stations V1 (33.23 ± 8.30 fmol cell^−1^ or 121.72 ± 7.05 nmol·mg^−1^ protein; equivalent to 3.95 ± 0.25 *μ*mol·g^−1^ FW) and V2 (34.78 ± 8.69 fmol cell^−1^ or 114.51 ± 7.69 nmol·mg^−1^ protein; equivalent to 4.54 ± 0.18 *μ*mol·g^−1^ FW). Increased levels of *p*CO_2_ also had a significant effect on the SOD activity in the anemone tentacles. In both, host (*F*_2, 27_ = 4.07, *P *=* *0.028) and zooxanthellae fractions (*F*_2, 27_ = 3.36, *P *=* *0.049), the SOD activity was significantly lower in tentacles from station V1 (host: 7.84 ± 1.37; zooxanthellae: 2.84 ± 0.41 U·mg^−1^ protein) compared with the control station (host: 13.19 ± 1.42; zooxanthellae: 4.72 ± 0.57 U·mg protein; Fig. 3).

## Discussion

Many experimental approaches to quantify the effects of increasing seawater *p*CO_2_ on the physiology and performance of organisms suffer from a lack of adequate exposure times. In contrast, natural CO_2_ vents, such as the one used in this study, provide unique experimental settings to assess the adaptive capacities of organisms to long-term increase in *p*CO_2_/decrease in pH at different biological levels (Hall-Spencer et al. 2008; Fabricius et al. 2011; Suggett et al. 2012).

Elevated *p*CO_2_ had no effect on chl a and zooxanthellae densities in *A. viridis*. This is in agreement with earlier observations by Towanda and Thuesen (2012) who found no significant changes in zooxanthellae densities or chl *a* concentrations in *Anthopleura elegantissima* following exposure to ˜2270 *μ*atm *p*CO_2_ under laboratory conditions. By contrast, a recent study on *A. viridis* along the CO_2_ gradient at Vulcano reported significantly higher zooxanthellae densities in animals growing under high *p*CO_2_ conditions compared with the intermediate and control sites (Suggett et al. 2012).

This discrepancy in results is likely the result of methodological differences in the quantification of algal cells. While Suggett et al. (2012) expressed zooxanthellae densities as cells per surface area of tentacle, cell numbers in our study and that by Towanda and Thuesen (2012) were normalized to mg of protein. This makes the comparison of results difficult. The handling of anemones greatly influences tentacle contraction, which may subsequently have led to a bias in surface area determination and thus an underestimation of tentacle surface area at station V1. This could also explain why the average zooxanthellae densities along the gradient reported by Suggett et al. (2012) were about an order of magnitude lower than those determined in this study.

The lack of differences in protein concentrations in the tentacles of *A. viridis* between stations indicated that *A. viridis* was in fact well acclimated to the high seawater *p*CO_2_ at this site (e.g., Langenbuch and Pörtner 2003). In contrast, DMSP_t_ concentrations were significantly higher under control *p*CO_2_ conditions than at elevated *p*CO_2_ at stations V1 and V2. This is consistent with the results from earlier studies showing increased cellular DMSP and DMS concentrations in response to CO_2_ limitation in different phytoplankton species (Sunda et al. 2002) and, at increased CO_2_, a decrease in DMS concentration during a mesocosm experiment (Hopkins et al. 2010) and DMS production in *E. huxleyi* laboratory cultures (Arnold et al. 2013). Increased DMSP concentrations within different algal species were also observed in response to UV radiation, temperature, and nutrient (N, Fe) limitation, factors that can cause oxidative stress (Sunda et al. 2002; Harada et al. 2009; McLenon and DiTullio 2012). DMSP concentrations in anthozoans can vary significantly with *Symbiodinium* clade (Steinke et al. 2011), which in turn may change within the host depending on their environmental optima (LaJeunesse et al. 2010). However, anemones in the Mediterranean commonly feature zooxanthellae belonging to clade A only (Savage et al. 2002; Visram et al. 2006). Our results corroborate this and additionally showed that zooxanthellae in anemonies of all three stations belonged to the same ITS2 “type”A19, as was previously demonstrated for *A. viridis* in Levante Bay by Suggett et al. (2012). This supports the notion that the differences in DMSP_t_ concentrations between stations were indeed influenced by ambient CO_2_ conditions and not the result of the genetic makeup of the zooxanthellae.

Previous studies reported enhanced photosynthetic rates of *A. viridis* at station V1 (Suggett et al. 2012) and of *A. elegantissima* following exposure to increased *p*CO_2_ under laboratory conditions (Towanda and Thuesen 2012), suggesting that elevated *p*CO_2_ with proximity to the vent site alleviated CO_2_ limitation, increased photosynthetic efficiency, and thereby decreased the generation of ROS (Asada 1994; Suggett et al. 2008). The notion that anemones exposed to elevated *p*CO_2_ experienced less oxidative stress than under control *p*CO_2_ is further supported by the low SOD activity in tentacles from station V1. DMSP_t_ concentrations normalized to both cell and protein content appeared to be more sensitive to increased *p*CO_2_ than SOD, showing a significant decrease at station V2 relative to the control, whereas the decrease in SOD activity toward higher *p*CO_2_ was gradual and significantly different only for station V1 relative to the control. This may be attributed to the specific phenotypic plasticity of DMSP and SOD in response to environmental cues (Ross and Van Alstyne 2007). DMSP_t_ concentrations may be thus enhanced only below a certain *p*CO_2_ threshold or above a given accumulation of ROS when the capacity of resident ROS scavenging systems is exceeded (Shen et al. 1997). In addition to enhanced photosynthesis, Suggett et al. (2012) observed increased growth of *A. viridis* in proximity to station V1, suggesting a link between growth and CO_2_-mediated stimulation of metabolism. Against the background of our results, it is also conceivable that enhanced growth of *A. viridis* was facilitated by a decreased energy investment in to the synthesis of antioxidant compounds such as SOD or DMSP.

In summary, while our data support the notion of an antioxidant functional role for DMSP in symbiotic noncalcifying anthozoans, more work is required to establish an explicit link between oxidative stress and DMSP dynamics in symbiotic cnidarians. Sea anemones are dominant organisms in many tropical and temperate coastal environments (e.g., Manuel 1981; Russo et al. 1994; Venn et al. 2008). The apparent sensitivity of DMSP_t_ concentration in *A. viridis* to *p*CO_2_ thus indicated that a doubling in current seawater *p*CO_2_ by the end of the century (Caldeira and Wickett 2005) could significantly decrease cellular DMSP_t_ concentration in these animals, which may further result in a decreased supply of DMSP and DMS to future coastal ecosystems.
